# AKR1B10 Inhibitor Epalrestat Facilitates Sorafenib-Induced Apoptosis and Autophagy Via Targeting the mTOR Pathway in Hepatocellular Carcinoma

**DOI:** 10.7150/ijms.42956

**Published:** 2020-05-18

**Authors:** Nan Geng, Yuanyuan Jin, Yurong Li, Shixuan Zhu, Han Bai

**Affiliations:** 1 Department of Infectious Disease, Shengjing Hospital of China Medical University, Shenyang 110004, China; 2 Laboratory of Biomaterials and Translational Medicine, The Third Affiliated Hospital, Sun Yat-Sen University, Guangzhou 510630, China

**Keywords:** hepatocellular carcinoma, AKR1B10, sorafenib, apoptosis, autophagy, mTOR signalling pathway

## Abstract

Sorafenib is the standard systemic treatment for advanced hepatocellular carcinoma (HCC), and improving its therapeutic effects is crucial for addressing cancer aggression. We previously reported that epalrestat, an aldo-keto reductase 1B10 inhibitor, enhanced sorafenib's inhibitory effects on HCC xenograft in nude mice. This study aimed to elucidate the mechanism of epalrestat's anti-tumour enhancing effects on sorafenib. HepG2 cells were treated with sorafenib, epalrestat, and their combination. Cell proliferation was assessed with Cell Counting Kit-8 and colony formation assays. AKR1B10 supernate concentration and enzyme activity were detected by ELISA assay and the decrease of optical density of NADPH at 340 nm. Cell cycle and apoptosis analyses were performed with flow cytometry. Western blots clarified the molecular mechanism underlying effects on cell cycle, apoptosis, and autophagy. The anti-tumour mechanism was then validated *in vivo* through TUNEL and immunohistochemistry staining of HCC xenograft sections. Epalrestat combined with sorafenib inhibited HepG2 cellular proliferation *in vitro*, arrested the cell cycle at G0/G1, and promoted apoptosis and autophagy. Treatment with a specific mTOR activator MHY-1485 increased mTOR phosphorylation, while suppressing apoptosis and autophagy. Consistent with *in vitro* results, data from the HCC-xenograft nude mouse model also indicated that combined treatment inhibited the mTOR pathway and promoted apoptosis and autophagy. In conclusion, epalrestat heightens sorafenib's anti-cancer effects via blocking the mTOR pathway, thus inducing cell cycle arrest, apoptosis, and autophagy.

## Introduction

Hepatocellular carcinoma (HCC) is a highly aggressive malignant tumour and the fourth leading cause of cancer-related death worldwide 1. Due to synergistic effects of hepatitis B and C infection, obesity, and alcohol consumption, HCC incidence is steadily rising 2. Survival rate of advanced HCC is extremely poor because of lower differentiation, insidious onset, rapid cancer recurrence, and low sensitivity to chemotherapy drugs 3. Sorafenib, a tyrosine kinase inhibitor, is the standard systemic treatment for advanced HCC 4. The drug primarily inhibits angiogenesis and tumour development through targeting vascular endothelial growth factor and the Raf/MAPK signalling pathway 5. However, the clinical outcome in patients with advanced HCC is far from satisfactory, indicating a crucial need for improving sorafenib's therapeutic effect.

The aldo-keto reductase family 1 member B10 (AKR1B10) is a member of the aldo-keto-reductase superfamily, normally expressed in small and large intestines 6. The protein converts carbonyl compounds on aldehydes or ketones to alcohols for detoxification and promotes de novo synthesis of fatty acids, regulating retinoic-acid metabolism and promoting cell proliferation. Highly expressed in liver cancer 7, AKR1B10 is closely related to clinical staging, HCC prognosis 8, and sensitivity to chemotherapeutic drugs 9. Our previous clinical studies showed that elevated serum AKR1B10 was related to diagnosis of early-stage HCC^10^, ^11^. Additionally, experimental studies in nude mice revealed that when combined with the AKR1B10 inhibitor epalrestat, sorafenib's inhibitory effects on HCC xenograft were enhanced 12. However, the mechanism of this enhancement remains unclear.

In this study, we performed *in vitro* and *in vivo* experiments to investigate the mechanism underlying epalrestat-induced enhancement of sorafenib's anti-tumour effects. Our work should provide a new therapeutic target for the treatment of patients with advanced HCC.

## Materials and Methods

### Cell culture

Human hepatocyte L02 cell line and HCC cell lines including HepG2, Huh-7 and PLC/PRF/5 were purchased from the Type Culture Collection of the Chinese Academy of Sciences (Shanghai, China). L02, HepG2 Cells were cultured in RPMI-1640 medium and Huh-7, PLC/PRF/5 cells were cultured in high glucose DMEM medium supplemented with 10% foetal bovine serum (FBS) and 1% penicillin-streptomycin (Biological Industries, Kibbutz Beit Haemek, Israel) in a 5% CO2, humidified atmosphere at 37°C.

### Chemicals and antibodies

Sorafenib (CAS, 284461-73-0), epalrestat (CAS, 82159-09-9) (Selleck Chemicals, Houston, TX, USA) and MHY-1485(CAS, 326914-06-1) (MedChem Express, Monmouth Junction, NJ, USA) were dissolved in dimethyl sulfoxide (Sigma-Aldrich, St. Louis, MO, USA) to produce 40 ,20 and 10 mmol/L stock solutions, respectively. Solutions were stored at -20°C. Rabbit monoclonal antibodies against phosphorylated retinoblastoma protein (p-Rb Ser807/811), cyclin D1, cyclin E1, caspase-3, cleaved caspase-3, B-cell lymphoma-2 (Bcl-2), extracellular regulated kinase (ERK), p-ERK (Tyr202/204), protein kinase B (AKT), p-AKT (Ser473), and mammalian target of rapamycin (mTOR) were purchased from Cell Signalling Technology (Danvers, MA, USA). Rabbit monoclonal antibodies against p-mTOR (S2448), BCL2-Associated X (Bax), Bcl-2-interacting protein-1 (Beclin-1), and microtubule-associated protein light chain-3 (LC3) were from Abcam (Cambridge, MA, USA). Rabbit monoclonal antibodies against AKR1B10 were from Invitrogen (Carlsbad, CA, USA). Rabbit monoclonal antibodies against GAPDH, β-actin, and β-tubulin were from Wanleibio (Shenyang, China).

### Cell viability assay

A Cell Counting Kit-8 assay (CCK-8; Do Jindo Molecular Technologies Inc., Kumamoto, Japan) was used to determine cell proliferation post-drug treatment. HepG2 cells were seeded in a 96-well plate (5 × 10^3^ cells/well) and cultured in 100 µL of RPMI-1640 supplemented with 10% FBS for 24 h. Next, cultures were replaced with different concentrations of epalrestat, sorafenib, and their combination. After incubation at 37°C for 24, 48, or 72 h, the medium was replaced with 90 µL of RPMI-1640 and 10 µL of CCK-8 reagent. Cells were incubated for 2 h at 37°C. Finally, optical density was measured using a microplate reader (elx808; BioTek Instruments Inc., Winooski, VT, USA) at 450 nm.

### Colony formation assay

The HepG2 cells were seeded into a six-well plate at 10^3^ cells/well in RPMI-1640 medium with 10% FBS and incubated for 24 h. Subsequently, 75 µmol/L epalrestat, 8 µmol/L sorafenib, and their combination was added to the cultures. After a 14-d incubation, cells were washed three times with PBS, fixed with 4% paraformaldehyde for 20 min, and stained for 5 min using crystal violet (Beyotime Institute of Biotechnology, Shanghai, China). The remaining dye was removed with ddH2O. Finally, colonies including >50 cells were counted to determine colony formation rate [(number of clones/number of cells inculcated) × 100%].

### ELISA assay of Supernates concentration of AKR1B10

After treated with 8 µmol/L sorafenib, 75 µmol/L epalrestat and the combination for 24h, cell culture supernates were collected to detect AKR1B10 concentration by enzyme-linked immunosorbent assay (ELISA) kits (USCN, Wuhan, China). According to the manufacturer's instructions, samples were centrifuged for 20 minutes at 1,000×g, followed by adding 100μL each of dilutions of standard and samples into the appropriate wells and incubating for 1 hour at 37 ℃. Then added Detection Reagent A, Detection Reagent B, Substrate Solution, and Stop Solution respectively. Finally, optical density was measured using a microplate reader (BioTek Instruments Inc., Winooski, VT, USA) at 450 nm.

### AKR1B10 enzyme activity assay

The reductase activities in the cell extracts were determined by the decrease in the absorbance of nicotinamide adenine dinucleotide phosphate (NADPH) at 340nm (*ε*_340_ = 6220 M^-1^ cm^-1^). Cells treated with 8 µmol/L sorafenib, 75 µmol/L epalrestat and the combination were lysed on ice in radioimmunoprecipitation buffer, supplemented with 1% PMSF, followed by centrifugation at 13000rpm at 4℃ for 20 minutes. The reaction mixture consisted of phosphate buffer saline (PH 7.0), 20mM DL-glyceraldehyde, 0.3mM NADPH and 50μg Soluble proteins in a total volume of 150µL. The decrease in optical density at 340 nm is monitored for 20 min at 37℃ in a microplate reader (BioTek Instruments Inc., Winooski, VT, USA) at 340 nm. One unit (U) of enzyme activity was defined as the amount of enzyme that catalyzes the oxidation of 1 μM NADPH per minute at 37 °C 13.

### Apoptosis and cell-cycle analysis

Cell apoptosis was determined using an Annexin V/propidium iodide (PI) apoptosis detection kit (BD Biosciences, Sunnyvale, CA, United States). The total apoptotic rate was characterized as the percentage of cells with Annexin V positive for early apoptosis and both Annexin V/propidium iodide (PI) positive for late apoptosis. HepG2 cells were seeded in six-well plates at 2 × 10^5^ cells/well. After 24 h of incubation, drugs (75 µmol/L epalrestat, 8 µmol/L sorafenib, or combination) were added to the culture medium for another 24 h of incubation, followed by staining for 15 min with Annexin V-FITC and PI in darkness. For cell-cycle analysis, treated cells were fixed with 70% ethanol for 12 h at 4°C before being washed three times with pre-cooled PBS. Cells were stained with RNase A for 30 min at 37°C and with PI (Solarbio, Beijing, China) for 15 min at 4°C, both in darkness. Apoptosis and DNA content were quantified using the BD FACScalibur flow cytometer.

### Immunofluorescence detection of p-Rb and LC3

HepG2 cells were cultured on glass coverslips to a suitable density before the addition of 75 µmol/L epalrestat, 8 µmol/L sorafenib, or their combination for 12 h. Cells were fixed for 20 min with 4% paraformaldehyde and permeabilised with Triton X-100 (0.2%) in PBS for 15 min. Unspecific binding sites were blocked with goat serum for 30 min. Coverslips were incubated with primary antibody against LC3 (1:200 diluted, Abcam) or p-Rb (1:400 diluted, CST) overnight, followed by incubation with fluorescein isothiocyanate (FITC)-labelled anti-rabbit IgG antibody (Abcam) for 1 h at room temperature. Cell nuclei were stained with 4ʹ,6-diamidino-2-phenylindole (DAPI) for 5 min. A confocal laser scanning microscope (Nikon, Japan) was used for image capture.

### Western blots

Cells were treated with 8 µmol/L sorafenib, 75 µmol/L epalrestat, or combination of 75 µmol/L epalrestat and 8 µmol/L sorafenib for 24h. For rescue experiment, cells were treated with 10µM mTOR activator MHY-1485 for 6h prior to treatment with the combination of sorafenib and epalrestat. Treated cells were lysed in radioimmunoprecipitation assay lysis buffer (Beyotime Institute of Biotechnology, Haimen, China), supplemented with 1% phenylmethanesulfonylfluoride (PMSF) and 1% phosphatase inhibitor cocktail. The mixture was centrifuged at 13,800 × *g* at 4°C for 20 min. Each well contained 25 μg of protein in a total volume of 10 μl. Proteins were visualised using chemiluminescence reagents (GE Healthcare Life Sciences, Pittsburgh, PA, USA) and detected with a chemiluminescence detection system (GE Amersham Imager 600). Protein bands were quantitated in ImageJ software.

### Immunohistochemistry (IHC) staining

Tumour tissues were obtained from previous animal experiments 12, all approved by the Animal Experimental Ethics Committee of the Shengjing Hospital of China Medical University (project identification code: 2017PS332K). Briefly, 1×10^7^ HepG2 cells were resuspended in 100ul culture medium and 100ul BD Matrigel, and the cell suspension were injected into the right side neck of nude mice. One week later, the mice were randomly divided into four groups: control, sorafenib (30 mg/kg), epalrestat (100 mg/kg), and combination (30 mg/kg sorafenib and 100 mg/kg epalrestat) group. The drugs were admitted by gavage, 6 times/week for two weeks. The mice were then sacrificed, and tumours were fixed. Paraffin sections were deparaffinised in xylene and rehydrated in ethanol and water. Sections were stained according to the SPlink Detection kit protocol (Zhongshan Golden Bridge Biotechnology Co. Ltd., Beijing, China), then incubated overnight at 4°C with primary antibodies against LC3 (1:200 dilution) or p-mTOR (1:100 dilution) for 30 min, followed by incubation with biotinylated anti-rabbit antibody and streptavidin/horseradish peroxidase at 37°C. Peroxidase reaction proceeded with 3,3-diaminobenzidine as the substrate; nuclei were stained with haematoxylin for 1 min. Images were then captured with a light microscope (Nikon, Japan); five images/samples were analysed using NIS-Elements Br3.0 software.

### TUNEL assay

In situ detection of apoptotic cells was performed with a TUNEL assay kit, following the manufacturer's protocol (Zhongshan Golden Bridge Biotechnology Co. Ltd.). Sections were deparaffinised in xylene and rehydrated in ethanol and water. Cell nuclei were stained with DAPI for 5 min. Images were then captured with a light microscope (Nikon, Japan); five images/samples were analysed using NIS-Elements Br3.0 software.

### Statistical analysis

All experiments were repeated independently three times and data were shown as means ± standard deviation. Statistical analyses were performed using SPSS 24.0 and significant differences were analysed by One-way analyses of variance (ANOVA) coupled with post hoc comparisons. Significance was defined as *P* < 0.05.

## Results

### Epalrestat enhances the effect of sorafenib on proliferation inhibition and cell-cycle arrest in HCC

**Figure 1:**
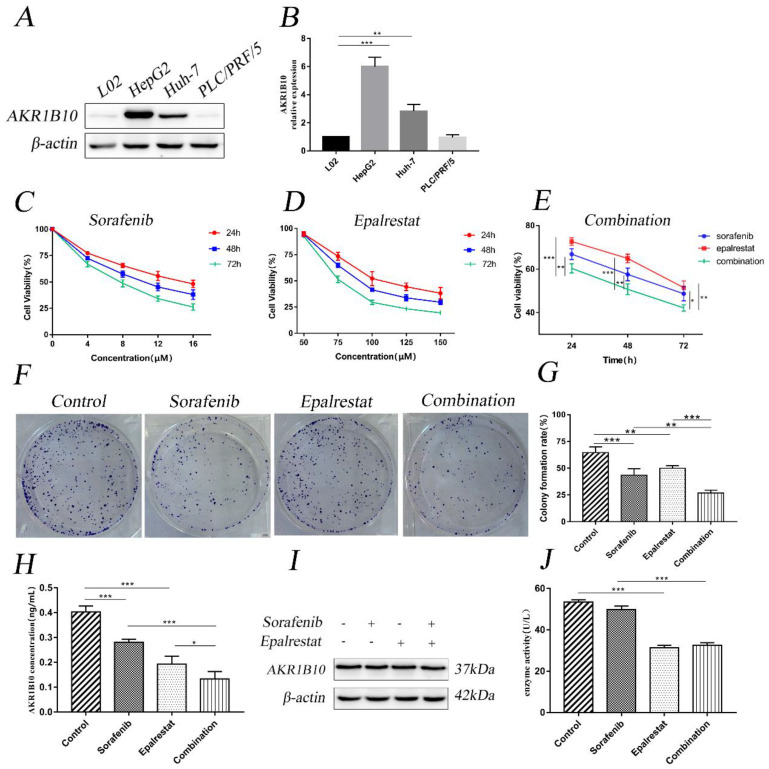
** Combination treatment of sorafenib and epalrestat inhibited HepG2 proliferation.** (A, B) Western blot was used to test the expression of AKR1B10 in hepatocyte L02 and HCC cell lines including HepG2, Huh-7 and PLC/PRF/5. (C-E) CCK-8 assay was used to detect HepG2 cellular viability after treatment with various concentrations of sorafenib (4, 8, 12 and 16 μM), epalrestat (50, 75, 100, 125 and 150 μM) and combination of 8μM sorafenib and 75μM epalrestat for 24, 48 and 72 h in HepG2 cells. (F, G) Colony formation assay of HepG2 cells in the single-agent or combination treatment. After 24h treatment with 8 μM sorafenib, 75 μM epalrestat

Western blot assay was used to detect AKR1B10 expression level in L02 and HepG2, Huh-7 and PLC/PRF/5 cell lines. We chose HepG2 cells with the highest AKR1B10 expression among the HCC cell lines for following studies (Figure 1A and B).

CCK-8 assay revealed that sorafenib inhibited HepG2 cell proliferation with half-inhibitory concentration (IC50) ranging between 8 and 16 µM (Figure 1C). However, epalrestat had no significant effect at low concentrations (Figure 1D). Notably, combining 8 µM sorafenib and 75 µM epalrestat significantly suppressed growth of HepG2 cells more than either monotherapy (Figure 1E). The colony formation assay revealed a comparable effect. After treatment with sorafenib or epalrestat for 14 d, HepG2 colony formation decreased significantly compared with control rates. Combined therapy reduced colony formation rate more than monotherapy (Figure 1F and G).

Since AKR1B10 is a secretory protein 14. Enzyme-linked immunosorbent assay (ELISA) was used to evaluate AKR1B10 levels in cell culture supernates. The assay showed epalrestat and sorafenib could inhibit AKR1B10 secret, while the combined therapy performed a higher effect than monotherapy (Figure 1H). Western blot assay revealed that the expression of AKR1B10 showed no change in HepG2 cells treated with sorafenib, epalrestat and the combination (Figure 1I). To determine the effect of epalrestat monotherapy and the combination on AKR1B10 activity, reduced NADPH at 340nm was used to measure AKR1B10 enzyme activity. The assay showed epalrestat could inhibit AKR1B10 enzyme activity, while no differences were identified in sorafenib compared with the untreated cells. Further, the combined therapy showed no differences between the epalrestat monotherapy (Figure 1J).

**Figure 2:**
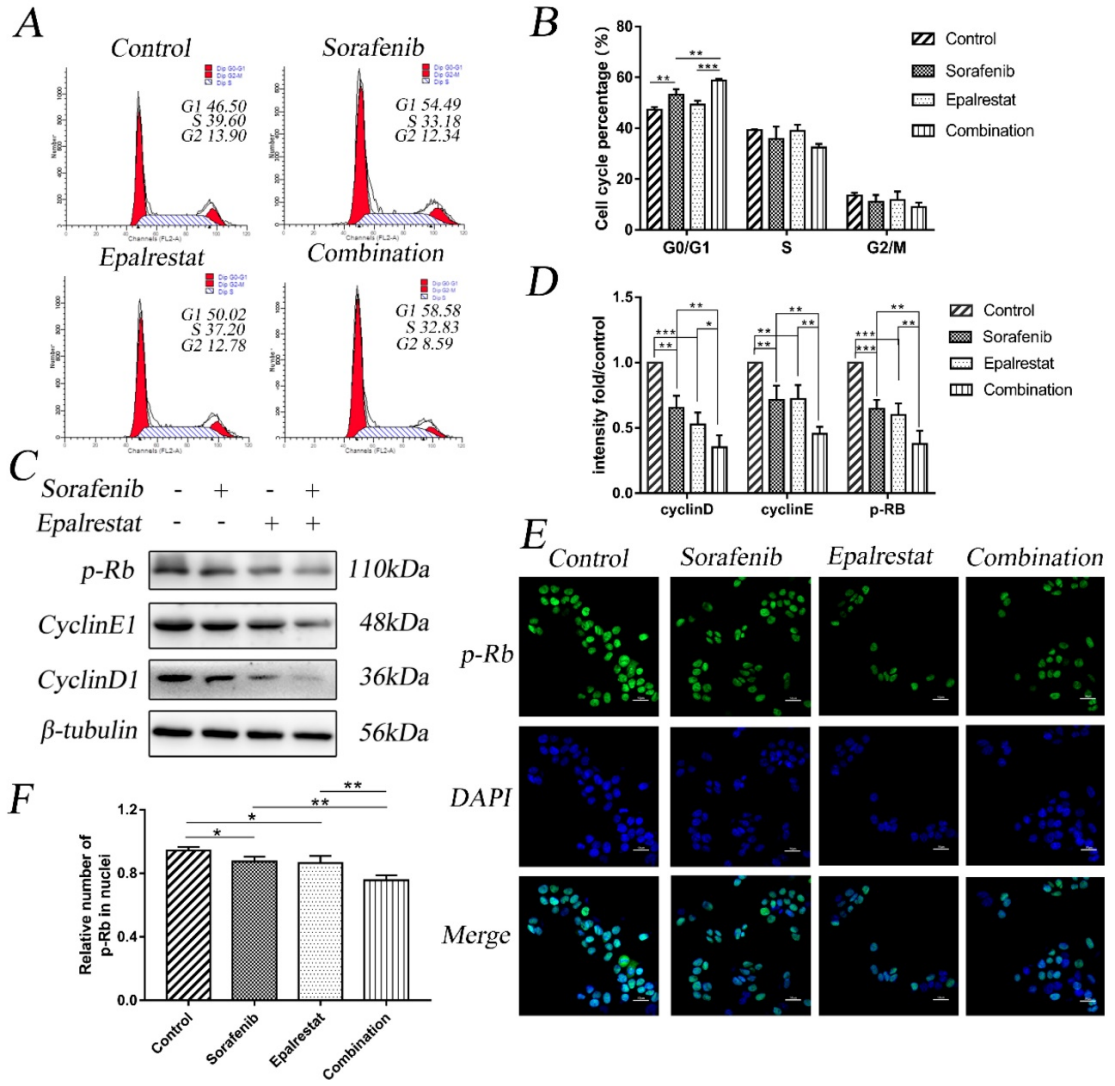
** Combination treatment of sorafenib and epalrestat induced G0/G1 cell-cycle arrest of HepG2 cells.** (A, B) cell cycle analysis using flow cytometry on HepG2 cells treated with 8 μM sorafenib, 75 μM epalrestat, and their combination for 24h. (C, D) Western blots of p-Rb, cyclin D1, and cyclin E1 after 24 h treatment with 8 μM sorafenib, 75 μM epalrestat, and combination. (E, F) Immunofluorescence assay detected nuclear p-Rb expression (400×). Data are mean ± SD. *, P < 0.05; **, P < 0.01; ***, P < 0.001.

Cell-cycle progression determines tumour proliferation 15. Here, sorafenib monotherapy increased the percentage of G1 cells compared with that in the control (53.18±2.22 vs 47.23±1.10, *P*=0.001). Epalrestat treatment and control did not differ (49.21±1.61 vs 47.23±1.10, *P*=0.146). However, combination therapy significantly increased the proportion of cells arrested at G0/G1 than sorafenib monotherapy (58.78±0.68 vs 53.18±2.22, *P*=0.002) (Figure 2A and B).

Cyclin, cyclin-dependent kinase (CDK), and transcription factor E2F1 regulate the cell cycle. Additionally, p-Rb specifically regulates the G1 to S transition. Our western blot results showed that while sorafenib and epalrestat monotherapy reduced the expression of cyclin D1, cyclin E1, and p-Rb, combination therapy had a stronger inhibitory effect on all three proteins (Figure 2C and D). Immunofluorescence assay identified a significant reduction in nuclear p-Rb expression under combination treatment (Figure 2E and F). These results demonstrated that sorafenib and epalrestat together enhanced anti-proliferative effects through inducing G0/G1 cell-cycle arrest in HCC.

### Epalrestat promotes sorafenib-induced cell apoptosis in HCC

Apoptosis is impaired during progression toward malignancy, leading to excessive cell proliferation. Promoting cancer cell apoptosis is therefore a goal for anti-cancer therapy. After treated with sorafenib, epalrestat monotherapy or combination for 24h, Annexin V/PI staining revealed the total apoptotic rate of control, sorafenib, epalrestat monotherapy and combination were 8.33±0.65, 13.40±1.41, 10.99±0.59, 19.69±1.26 respectively. Both sorafenib and epalrestat monotherapy increased total HepG2 apoptotic rate compared with that in the control. Under combination treatment, total apoptotic rate was significantly higher than that of monotherapy.

**Figure 3:**
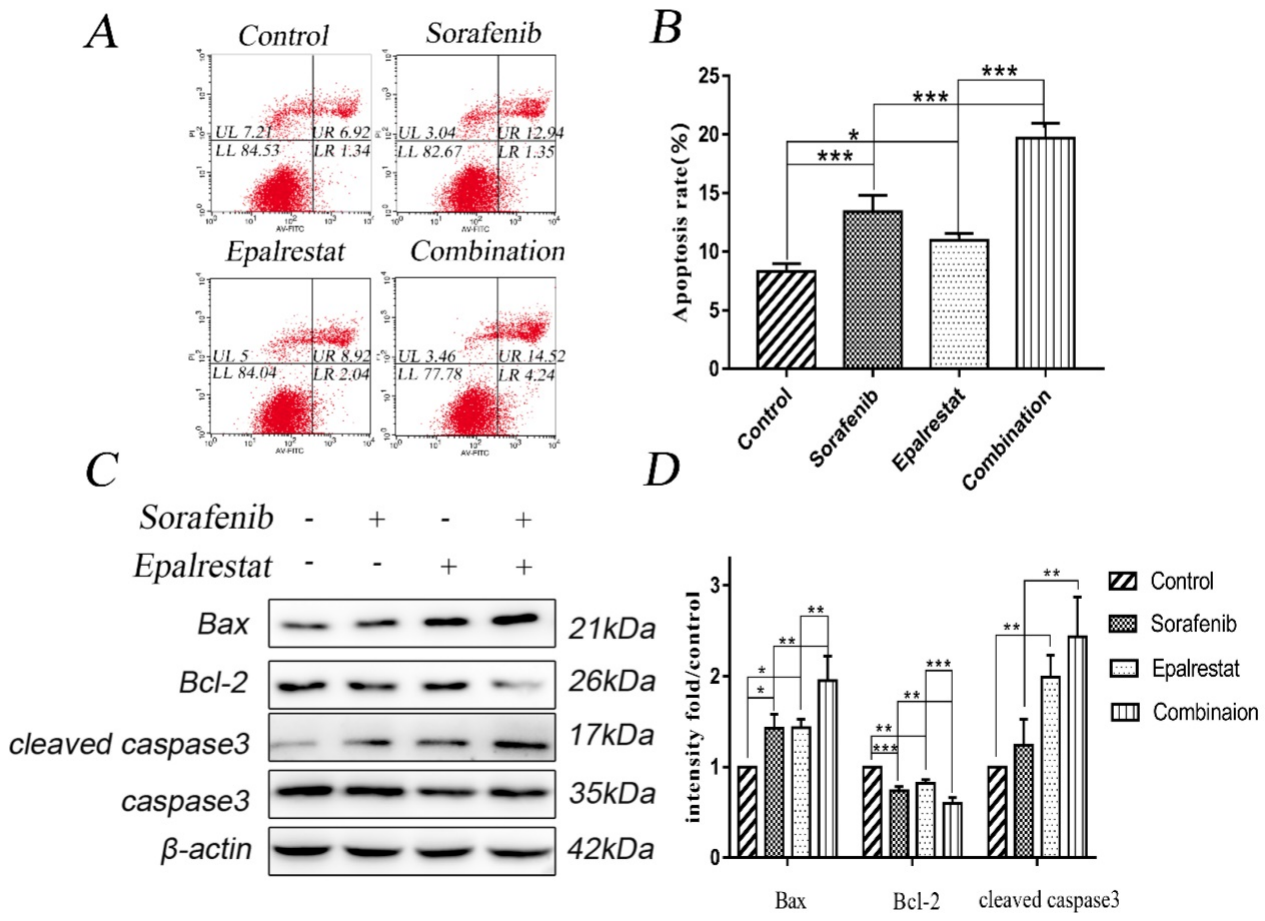
** Sorafenib and epalrestat combination promoted apoptosis of HepG2 cells.** (A, B) Apoptosis rate was evaluated with annexin V/PI staining. (C, D) Western blots of Bax, Bcl-2, caspase-3, and cleaved caspase-3, after treatment with 8 μM sorafenib, 75 μM epalrestat, and their combination for 24h. Data are mean ± SD. *, P < 0.05; **, P < 0.01; ***, P < 0.001.

Caspase-3 and its activated, cleaved form are apoptosis effectors. Additionally, pro-apoptotic protein Bax and anti-apoptotic protein Bcl-2 play key roles in maintaining apoptosis balance 16. Western blots revealed that both sorafenib and epalrestat up-regulated Bax and cleaved caspase-3 expression, while down-regulating Bcl-2 expression. Combination treatment increased both of these effects, thus enhancing apoptosis in HepG2 cells (Figure 3C and D).

### Epalrestat promotes sorafenib-induced cell autophagy in HCC

**Figure 4:**
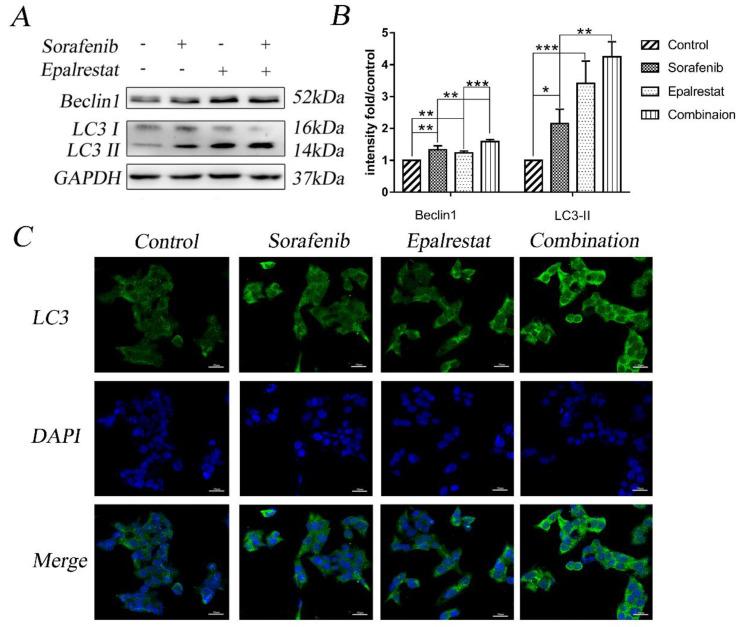
** Combination treatment of sorafenib and epalrestat activated autophagy of HepG2 cells.** (A, B) Treatment with 8 μM sorafenib, 75 μM epalrestat and the combination for 24 h. Western blot was applied to detect the levels of Beclin1 and LC3. (C) Immunofluorescence assay detected LC3 expression in cytoplasm (400×). Data are mean ± SD. *, P < 0.05; **, P < 0.01; ***, P < 0.001.

Multiple stimuli activate the processes of autophagy and apoptosis to regulate cell death 17. LC3-II is a specific marker for autophagy initiation 18; additionally, Beclin-1 complexation with Atg14 and VPS34 contributes to autophagosome nucleation 19. Here, western blot results showed that sorafenib and epalrestat monotherapy up-regulated Beclin-1 expression and promoted LC3 type I-to-type-II conversion, suggesting the activation of autophagy. Combination therapy significantly up-regulated Beclin-1 and LC3-II expression more dramatically than monotherapy (Figure 4A and B). Consistent with western blots, immunofluorescence also showed that LC3 was distributed in the cytoplasm, with significantly up-regulated expression under combined therapy (Figure 4C). These results reported that sorafenib and epalrestat combination activated autophagy in tumour cells.

### Sorafenib and epalrestat combination activates apoptosis and autophagy via inhibiting the mTOR pathway

**Figure 5:**
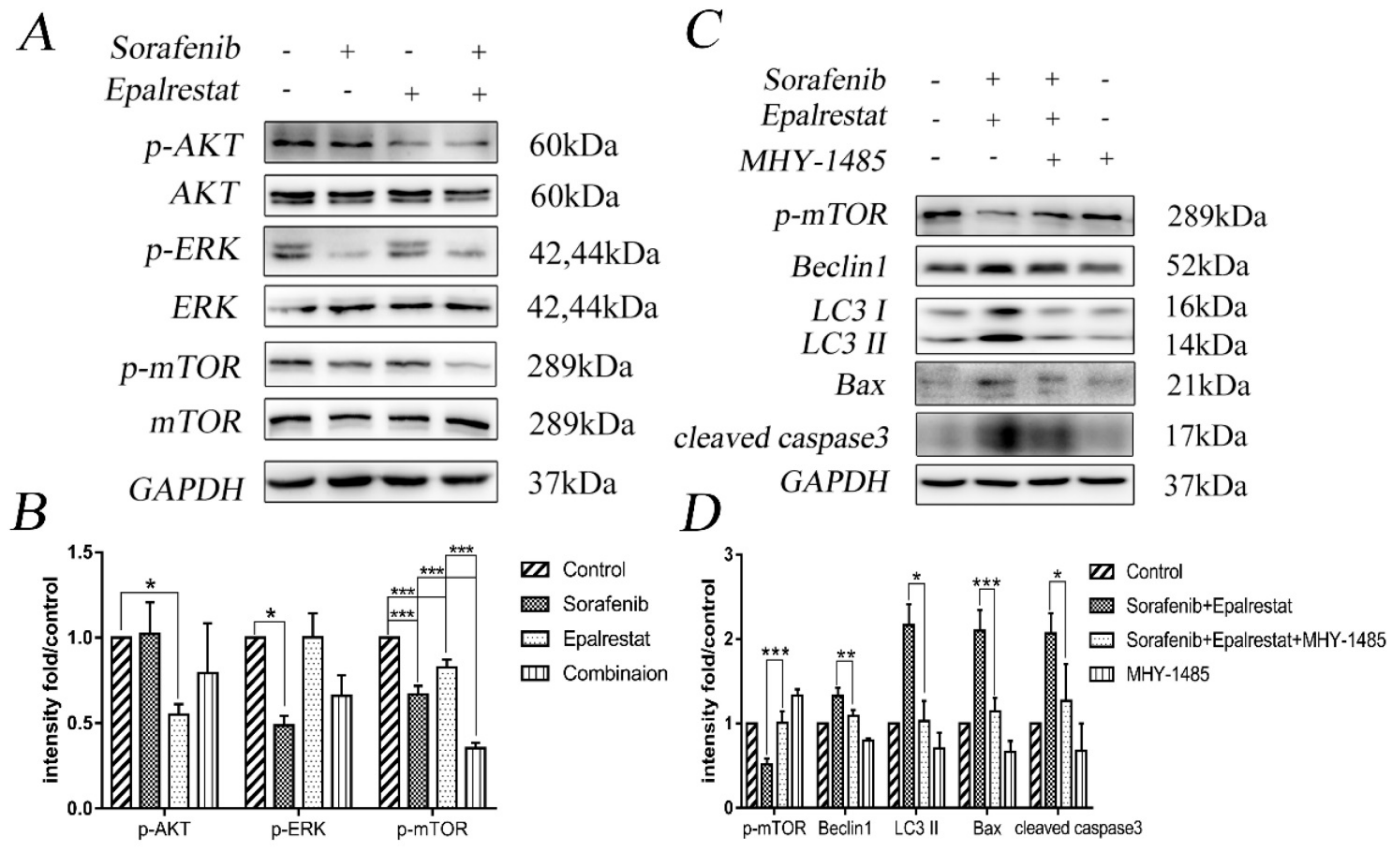
** Sorafenib and epalrestat combination activated autophagy and apoptosis through inhibiting the mTOR pathway.** (A, B) After 24h treatment with 8 μM sorafenib, 75 μM epalrestat, and their combination, western blots were applied to detect p-AKT, AKT, p-ERK (44/42), ERK (44/42), p-mTOR and mTOR. (C, D) HepG2 cells were treated with MHY-1485 for 6h prior to the combination treatment, western blots were applied to detect p-mTOR Beclin1, LC3 II, Bax, cleaved caspase3. Data are mean ± SD. *, P < 0.05; **, P < 0.01; ***, P < 0.001.

The serine/threonine kinase mTOR plays an important role in regulating autophagy, proliferation, and angiogenesis 20. The mTOR pathway is co-regulated through MAPK and AKT signalling pathways 21. Our western blots showed that sorafenib and epalrestat down-regulated p-ERK and p-AKT individually, while they both inhibited mTOR phosphorylation, with more significant inhibitory effect under combination treatment (Figure 5A and B).

To verify that the combination therapy induced apoptosis and autophagy via the mTOR signalling pathway, we treated HepG2 cells with the mTOR activator MHY-1485 prior to combined drug treatment. The results revealed that the combination increased the expression of Beclin-1, LC3 II, Bax, and cleaved caspase-3 while decreased the phosphorylation of p-mTOR compared with that of the control group. However, the combination-induced effect was significantly reversed by MHY-1485.These results further confirmed that sorafenib and epalrestat likely activate autophagy and apoptosis through targeting the mTOR pathway.

### Epalrestat and sorafenib combination reduces tumour growth in HCC xenograft model though targeting mTOR phosphorylation,

**Figure 6:**
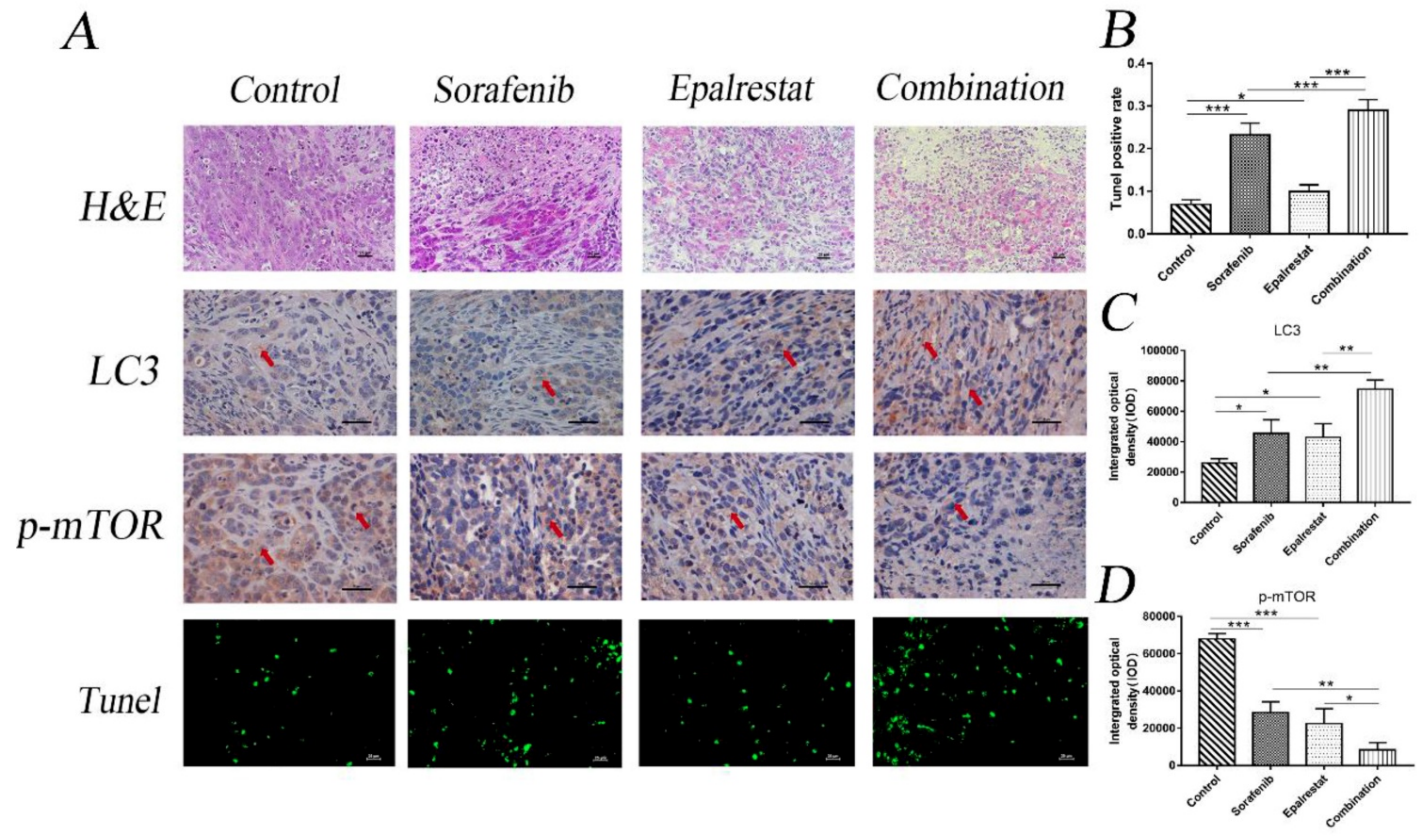
** Sorafenib and epalrestat combination promoted apoptosis, autophagy, and reduced mTOR phosphorylation in HCC xenograft tissue.** TUNEL assay and IHC staining of LC3 and p-mTOR were used to evaluate the levels of apoptosis, autophagy and p-mTOR in the combination treatment compared to monotherapy (400×). Data are mean ± SD. *, P < 0.05; **, P < 0.01; ***, P < 0.001.

Consistent with the in vitro results, TUNEL assays and LC3 staining in xenograft tumours also indicated that epalrestat facilitated sorafenib-induced apoptosis and autophagy (Figure 6A and B) (Figure 6A and C). Similarly, IHC staining revealed that sorafenib significantly inhibited p-mTOR phosphorylation, an effect enhanced under combination treatment (Figure 6A and D). These results further demonstrated that combination of epalrestat and sorafenib may promote apoptosis and activated autophagy by targeting the mTOR signalling pathway.

## Discussion

Sorafenib inhibits tumour angiogenesis and proliferation through targeting vascular endothelial growth factor receptor 2,3, platelet-derived growth factor receptor, and the MAPK signalling pathway 22. Sorafenib also inhibits the expression of cyclin D1, Mcl-1, and survivin, all of which are involved in cell-cycle arrest and apoptosis 23. Despite improvement in the survival benefit of advanced HCC by sorafenib, the median survival time of advanced HCC patients has only been extended by 3 months 4. Furthermore, some patients experience tumour progression during sorafenib therapy and show a low response rate. Most advanced HCCs acquire resistance to sorafenib due to hypoxic microenvironment, abnormal activation of PI3K/AKT, tumour-initiating cells, and epithelial-mesenchymal transition 24. Thus, exploring combined strategies is a potentially effective way to improve sorafenib therapeutic effects.

AKR1B10 is a secretory protein and regarded as potential serum marker of hepatocellular carcinoma. It has been reported that AKR1B10 is secreted through a lysosome-mediated nonclassical pathway, leading to an increase in the serum of cancer patients^14^. Epalrestat is the AKR1B10 inhibitor, which inhibits its secretion and enzyme activity rather than its expression. In this study, we found epalrestat can inhibit AKR1B10 enzyme activity and concentration in cell culture supernates. Sorafenib had little effect on AKR1B10 enzyme activity, but it could reduce AKR1B10 concentration in the supernates. It is suggested that the inhibition of AKR1B10 secretion and enzyme activity may be the mechanism of epalrestat enhancing the efficacy of sorafenib.

Deregulated cell cycle and increased telomerase activity cause sustained cell proliferation and genomic instability, both of which are characteristic features of carcinogenesis 15. Our results showed that combining sorafenib and epalrestat was more effective than monotherapy in significantly inhibiting cell proliferation and colony formation ability, as well as promoting G0/G1 cell-cycle arrest. We also demonstrated that monotherapy reduced cyclin D1, cyclin E1, and p-Rb expression, with combination therapy exerting a stronger inhibitory effect. All of these proteins may play an important role in cell proliferation and cell-cycle arrest. The Rb protein inactivates transcription factor E2F1, which plays an important role in cell proliferation, through masking its transactivation domain. After the cyclin-CDK complex is formed, Rb is phosphorylated and releases E2F1, thereby driving the cell cycle from G1 to S 25. Therefore, combination therapy enhanced anti-tumour effects through the mechanism of cell-cycle arrest.

Apoptosis maintains intracellular homeostasis, with the cascade activation of the caspase family playing a key role in initiation 16, while the Bcl-2 superfamily is important for regulation. Insertion of pro-apoptotic proteins Bax and Bak into the mitochondrial membrane releases cytochrome c into the cytoplasm. As a result, apoptotic bodies are synthesised to recruit and activate caspase-9 and -3, thus initiating apoptosis. Anti-apoptotic proteins Bcl2 and Bcl-xL anchor to the mitochondrial membrane through their c-terminals, preventing apoptosis through inhibiting mitochondrial membrane permeability and cytochrome c release 26. Because apoptosis plays an important role in tutor occurrence, development, and prognosis, anti-tumour therapy often targets apoptosis promotion 27. Our results revealed that compared with monotherapy, combination treatment significantly increased total HepG2 apoptotic rate, along with Bax and cleaved caspase 3 expression, while decreasing Bcl-2 expression. We confirmed that epalrestat promoted HCC apoptosis through regulating the Bcl-2/caspase-3 pathway, thus enhancing sorafenib anti-tumour effects.

Autophagy or type-II programmed cell death exerts a dual directional regulation effects on tumours. Occurring under stressors such as hypoxia or starvation, autophagic cells consume long-lived proteins or damaged organelles to maintain energy balance 28. When induced by chemotherapeutic drugs, autophagy is considered an anti-tumour mechanism 29. Sorafenib can induce autophagy in HCC 30, and sorafenib-resistant cells exhibit autophagy inactivation. Therefore, restoring autophagy is a therapeutic target for drug-resistant cancer cells 31. Previous studies have shown that inhibiting aldose reductase causes accumulation of lipid peroxides such as 4-hydroxynonenal and acetaldehyde protein adducts; this accumulation then results in autophagy activation 32. Here, we found that both sorafenib and epalrestat activated autophagy through increasing Beclin-1 and LC3 expression. Thus, enhanced autophagy appears to be a mechanism behind the improved therapeutic effects of combination therapy. Growing evidence has demonstrated that autophagy can regulate apoptosis through the mitochondrial pathway. Autophagy-related proteins also play an important role in apoptosis. Beclin-1 contains the Bcl-2 homology-3 domain, which can bind to bcl-2, acting as a link between autophagy and apoptosis.

**Figure 7:**
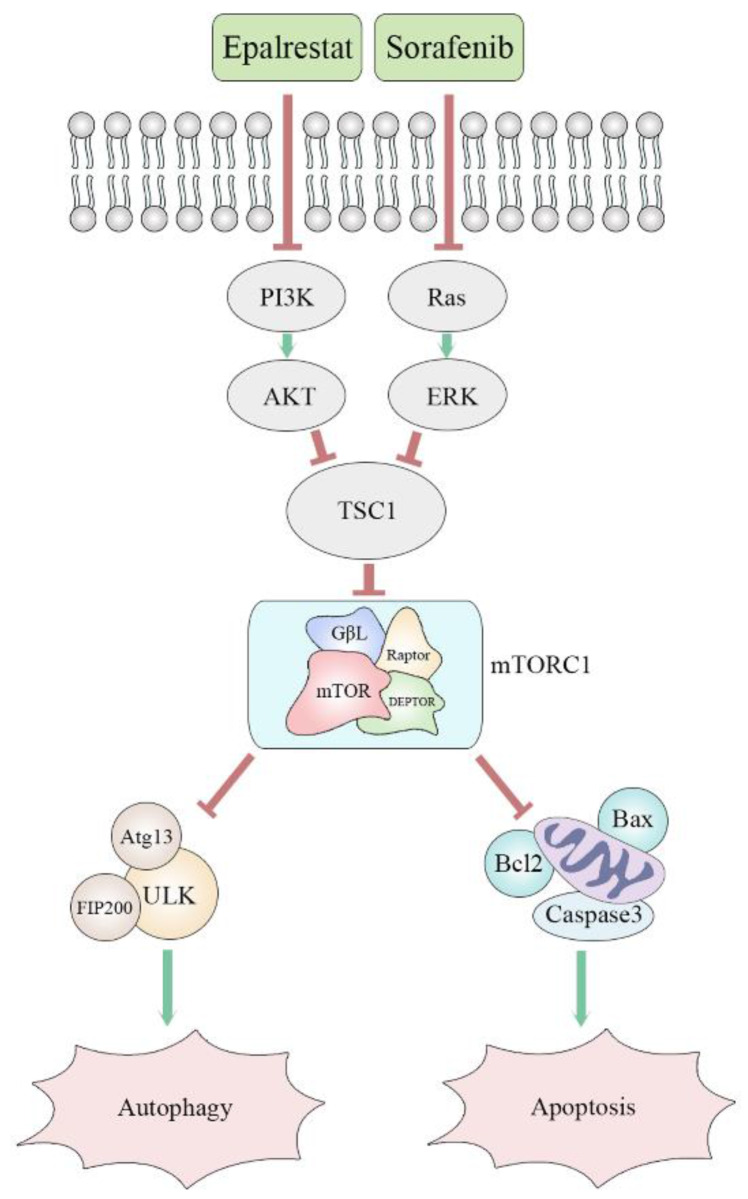
Proposed mechanism of synergistic effect of sorafenib and epalrestat on HCC.

Many cancer cells, including HCC, exhibit up-regulation of mTOR. The protein mTORC1 regulates cell proliferation, apoptosis, angiogenesis, and autophagy through targeting its downstream molecules, including S6K, 4EBP1, HIF-1alpha, and ULK1 33. Both ERK and AKT signalling pathways are involved in mTOR pathway regulation. Specifically, AKR1B10 targets ERK signalling to regulate lipid metabolism, a role that appears to be carcinogenic in many tumours. Previous studies reported that AKR1B10 increased the synthesis of the lipid second messengers PIP2 and DAG in breast cancer cells, activating the Raf/MEK/ERK signalling pathway and promoting cancer cell proliferation 34. However, in this study, epalrestat had no significant inhibitory effect on p-ERK, while sorafenib, as a MAPK inhibitor, had a significant inhibitory effect. The abnormal activation of the PI3K/AKT signalling pathway is linked to secondary sorafenib resistance. It is reported that aldose reductase inhibitor can promote G0/G1 cell cycle arrest of colon cancer cells by inhibiting PI3K/AKT/E2F1, and inhibit the proliferation and invasion by inhibiting PI3K/AKT/GSK3β 35, 36. Our results indicated that epalrestat inhibited p-AKT, while sorafenib had no significant effect. Both sorafenib and epalrestat decreased p-mTOR phosphorylation, an effect made stronger under combination therapy. Importantly, p-mTOR was found to be downregulated during combination treatment with sorafenib and epalrestat. However, MHY-1485, a mTOR activator, rescued the decreased level of p-mTOR. Moreover, increased phosphorylation of p-mTOR reduced the apoptosis and autophagy induced by the combination treatment. Clearly, the mTOR signalling pathway is involved in the mechanism of the effect of combination treatment (Figure 7).

In conclusion, epalrestat combined with sorafenib enhanced the latter's anti-tumour effects in HCC. The underlying mechanism was regulation of cyclin-CDK/p-RB-mediated G0/G1 cell-cycle arrest, bcl-2/caspase-3-mediated apoptosis, and inhibiting the mTOR pathway to activate autophagy. Our findings strongly suggest that this combination therapy is a potential treatment option against HCC.

## Abbreviations

AKR1B10: aldo-keto reductase 1B10
HCC: hepatocellular carcinoma
IHC: immunohistochemistry (IHC)
mTOR: mammalian target of rapamycin
CDK: cyclin-dependent kinase
LC3: microtubule-associated protein light chain3
p-Rb: phosphorylated retinoblastoma protein
Bcl-2: B-cell lymphoma-2
ERK: extracellular regulated kinase
AKT: protein kinase B
Bax: BCL2-Associated X
Beclin-1: Bcl-2-interacting protein-1 (Beclin-1)
CCK-8: Cell Counting Kit-8 assay
